# Synthesis and Supramolecular Structure of a (5-(3-(1*H*-tetrazol-5-yl)phenyl)-1*H*-tetrazole) Cobalt Complex

**DOI:** 10.1155/2010/104329

**Published:** 2010-06-15

**Authors:** George E. Kostakis, Christopher E. Anson, Annie K. Powell

**Affiliations:** ^1^Institute of Nanotechnology, Karlsruhe Institute of Technology, Postfach 3640, 76021 Karlsruhe, Germany; ^2^Institute of Inorganic Chemistry, Karlsruhe Institute of Technology, Engessertsrasse 15, 76131 Karlsruhe, Germany

## Abstract

The reaction of CoCl_2_ *·*6H_2_O with *m*-BDTH_2_ (1,3-benzeneditetrazol-5-yl) leads to [Co(C_8_H_6_N_8_)_2_(H_2_O)_2_(CH_3_CN)_2_]Cl_2_ (**1**). Both tetrazolic groups remain protonated existing in a 1*H* tautomeric form. A trifurcated chlorine atom and stacking interactions assemble compound **1** into a three-dimensional network.

## 1. Introduction

**Scheme 1:**
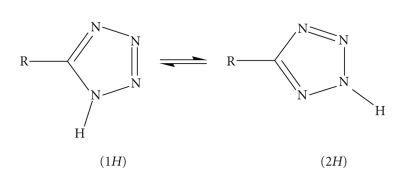
The two possible tautomers of 5-substituted tetrazoles.

Tetrazoles are a class of organic heterocyclic compounds which consist of a five-membered ring of four nitrogen atoms and one carbon atom (Scheme 1). In the design of drug molecules, tetrazoles and tetrazole derivatives have generally been avoided because their explosive and flammable nature makes them a safety concern for process-scale synthesis. Nevertheless, tetrazoles such as Losartan [1] and Candesartan [2] are angiotensin II receptor antagonist drugs used mainly to treat high blood pressure (hypertension). Moreover, a well-known tetrazole is dimethyl thiazolyl diphenyl tetrazolium salt (MTT), which is used in the MTT assay of the respiratory activity of live cells in cell culture [3]. 5-substituted-1*H*-tetrazoles (RCN_4_H) usually can be utilised as metabolism-resistant isosteric replacements for carboxylic acids (RCO_2_H) in SAR-driven medicinal chemistry analogue syntheses, while it has been found that they have comparable pKa values to the corresponding carboxylic acids (RCO_2_H) [4]. Therefore, the study of the structures of tetrazoles is relevant to several aspects of medicinal chemistry, as, indeed, is their coordination chemistry given the increasing importance of metal-based drugs incorporating known therapeutic organic agents [5].

5-substituted-1H-tetrazoles, often termed tetrazolic acids, can be found in neutral, anionic, or cationic form, and they can act as ligands in metal complexes, form salts, and can be both acceptors and donors for hydrogen bonding. Such tetrazolic acids exist in an approximately 1 : 1 ratio of the 1*H*- and 2*H*-tautomeric forms (Scheme 1). It has been reported that the two positional isomers 1 and 2 may be differentiated on the NMR timescale [6, 7], while theoretical calculations show that the 2*H*-tautomers are the more stable isomers, although they were found to have a larger degree of electron delocalization than 1*H*-tautomers [8]. Over the last decade, the synthetic procedures of such compounds have been improved [9, 10]; consequently more attention has been focused on the coordinating behaviour of such compounds. Thus, the 5-substituted-1*H*-tetrazoles have been used as versatile building blocks for molecular coordination networks, also called coordination polymers or metal organic frameworks [11, 12] as well as hydrogen bonded frameworks [13–16]. 

Recently, we embarked on a study of the coordination behaviour of various 5-substituted-1*H*-tetrazoles [13–16], and this article describes part of this systematic study where we report the synthesis and crystal structure of [Co(*m*-BDTH_2_)_2_(H_2_O)_2_(CH_3_CN)_2_]Cl_2_ (**1**) where *m*-BDTH_2_ = 1,3-benzeneditetrazol-5-yl. 

## 2. Experimental

### 2.1. General

All chemicals and solvents used for the synthesis were obtained from commercial sources and were used as received. The reaction was carried out under aerobic conditions. Elemental analysis (C, H, N) was performed at the Institute of Inorganic Chemistry, Karlsruhe Institute of Technology, using an Elementar Vario EL analyzer. Fourier transform IR spectra were measured on a Perkin-Elmer Spectrum One spectrometer with samples prepared as KBr discs.

### 2.2. Preparation of the Ligand and Compound (**1**)

*m*-BDTH_2_ was prepared as described previously [7]. A solution of *m*-BDTH_2_ (0.021 g, 0.1 mmol) in CH_3_CN/EtOH (ratio 1 : 1, total volume 20 ml) was added dropwise to a stirred solution of CoCl_2_ 6H_2_O (24 mg, 0.1 mmol). The resulting dark blue solution was refluxed for 2 hours, filtered and left to evaporate slowly. Orange crystals of [Co(C_8_H_6_N_8_)_2_(H_2_O)_2_(CH_3_CN)_2_]Cl_2_ (**1**) were formed after slow evaporation of the solution (Yield: 27 mg, 80%). Calc. for C_20_H_22_Cl_2_CoN_18_O_2_, (**1**): C, 35.55; H, 3.28; N, 37.34. Found C, 35.69; H, 3.31; N, 37.32%. IR (KBr, cm^−1^) = 3338 (s), 3053 (w), 2896 (m), 2846 (m), 2822 (m), 2737 (w), 2317 (m), 2290 (m), 1711 (w), 1621 (w), 1553 (s) 1481 (s), 1459 (s), 1374 (m), 1256 (m), 1169 (m) 1144 (w) 1127 (w) 1084 (s), 1029 (s) 999 (m) 906 (w), 872 (m) 802 (s), 762 (s), 733 (s), 705 (s), 684 (s). 

### 2.3. X-Ray Crystallography

Single-crystal X-ray crystallographic data of **1** were collected at 100 K on a Bruker SMART Apex CCD diffractometer using graphite-monochromated Mo-K*α* radiation. Crystallographic data and details of the measurement and refinement are summarized in Table 1. Semiempirical absorption corrections were made using SADABS [17]. The structures were solved using direct methods followed by full-matrix least-squares refinement against *F* ^2^ (all data) using SHELXTL [18]. Anisotropic refinement was used for all non-H atoms; all H atoms were refined (both coordinates and isotropic temperature factors) without restraints, except for the methyl H-atoms on the acetonitrile solvent molecules, which were refined as a rigid tetrahedral group, but with the torsional angle allowed to refine (AFIX 137 in SHELXTL). The crystallographic data and refinement parameters are listed in Table 1. 

## 3. Results and Discussion

### 3.1. Crystal Structure of **1**

**Figure 1:**
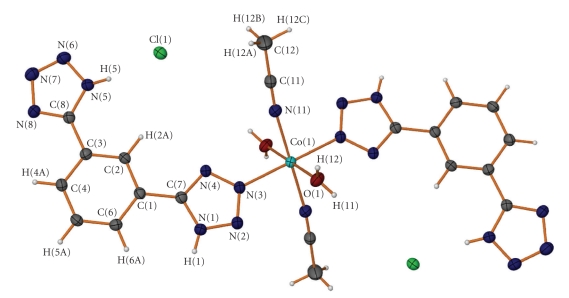
View of the cobalt complex in **1**, shown in approximately the same orientation, with the atom-labelling scheme. Ellipsoids represent displacement parameters at the 40% probability level.

Compound **1** was characterized crystallographically and found to belong to the triclinic *P*-1 space group. Selected distances (Å) and angles (°) are presented in Table 2. The Co(II) atom is six-coordinate with an octahedral geometry. The coordination sphere is occupied by two nitrogen N(3) atoms provided by two *m*-BDTH_2_ ligands, two acetonitrile, and two water molecules. Both tetrazolic groups remain protonated existing in a 1*H* tautomeric form. To our knowledge this is the first example showing the neutral form. Only one of the two aryl moieties is coordinated to the Co(II) atom through the nitrogen atom N3 (Figure 1). The dihedral angles between the planes through the coordinated tetrazole, the central aryl moiety, and the noncoordinated tetrazole are 5.87° and 9.8°, respectively.

**Figure 2:**
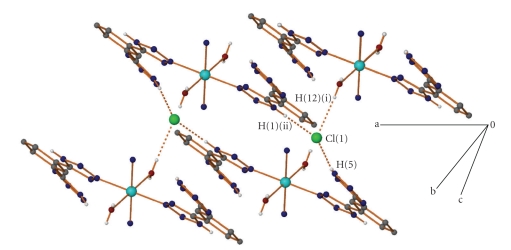
A projection of the hydrogen bonded layer formed in **1**, parallel to *a* axis. The organic hydrogen atoms and acetonitrile carbon atoms have been omitted for clarity.

**Figure 3:**
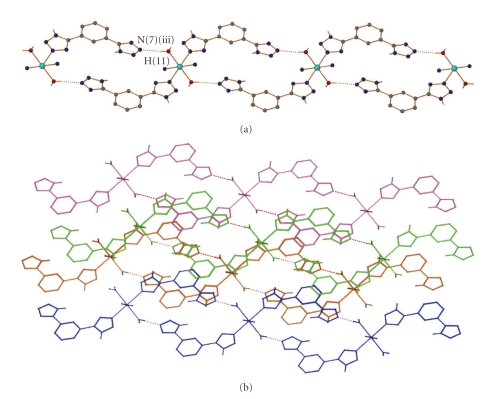
(a) A projection of the hydrogen bonded layer formed in **1** perpendicular to *a *axis; a view of **1** showing the *π* – *π* interaction. (b) The hydrogen-bonded layers are drawn in different colours to emphasize the stacking interactions between the aryl rings that lead to a 3D supramolecular architecture. Chlorine atoms, the organic hydrogen atoms, and acetonitrile carbon atoms have been omitted for clarity.

In compound **1 **there are two different kinds of interactions that lead to the formation of a 3D supramolecular architecture, namely, hydrogen bonding and stacking interactions. The chlorine atom is part of a trifurcated hydrogen bonding arrangement (Table 3) between three different cations. In this way a layer parallel to the *a* axis is formed (Figure 2). The first H-bond involves hydrogen atom H(5) of the noncoordinated tetrazolic group, the second involves hydrogen atom H(12) of the coordinated water molecule and the third involves hydrogen atom H(1) of the coordinated tetrazole group. In addition, a fourth hydrogen bond is located between hydrogen atom H(11) of water molecule and nitrogen atom N(7) of the noncoordinated tetrazolic group, forming an infinite 1D-chain perpendicular to the *a* axis. These 1D chains interact with each other through weak *π* − *π* stacking interactions involving all the aryl moieties (Table 3). A stronger interaction occurs between the central ring and the noncoordinated tetrazolic group, while there is also an interaction with an adjacent coordinated tetrazolic group belonging to a third 1D chain (Figure 3). 

### 3.2. FT–IR Spectroscopy

The IR spectrum of **1** displays broad bands centred at 3338 cm^−1^, in agreement with the presence of H-bonded water molecules. The bands at 2317 (m), 2290 (m), can be attributed to the *ν*(CN) of the acetonitrile while the bands at 1553, 1481,1459, 802, 762, 733, 705, and 684 cm^−1^ are characteristic of the nondeprotonated ligand [13].

## 4. Conclusions

In this article, we have examined the interaction of *m*-BDTH_2_ = 1, 3-benzeneditetrazol-5-yl with CoCl_2_·6H_2_O. The absence of any base in the reaction mixture prevents deprotonation of the ligand, and in addition to the fact that the ligand coordinates via the nitrogen atom at position 2 on the ring, the hydrogen atom on the nitrogen at position 1 could be located and refined giving clear evidence that the tetrazole is in the 1*H* tautomeric form (Scheme 1, left). Both the tetrazolic groups of the ligand remain protonated and in the 1*H* tautomeric form and, to our knowledge, this is the first example of a coordination compound with this neutral form. The trifurcated hydrogen bonding arrangement at the chloride counterion and stacking interactions assemble compound **1** into a three-dimensional supramolecular network. The present finding provides new structural data, which could enhance the understanding of the structural aspects of 5-substituted-1*H*-tetrazoles. This work represents a part of our systematic efforts to determine new synthetic pathways in the binary system metal/5-substituted-1*H*-tetrazoles, and further studies on the influence of other parameters on the hydrogen-bonded structural motifs are in progress.

## Figures and Tables

**Table 1:** Crystal data and structure refinement for **1**.

Empirical formula	C_20_H_22_Cl_2_CoN_18_O_2_	
Formula weight	676.39	
Temperature	100(2) K	
Wavelength	0.71073 Å	
Crystal system	Triclinic	
Space group	*P*-1	
Unit cell dimensions	*a* = 8.3341 (4) Å	*α* =84.682 (1)°
	*b* = 8.4696 (5) Å	*β* = 73.522 (1)°
	*c* = 11.4730 (6) Å	*γ* = 65.938 (1)°
Volume	708.85 (7) Å^3^	
Z	1	
Density (calculated)	1.584 Mg/m^3^	
Absorption coefficient	0.85 mm^−1^	
F(000)	345	
Crystal size	0.36 × 0.31 × 0.25 mm	
Index ranges	−10 ≤ h ≤10, −10 ≤ k ≤ 11, −14 ≤ l ≤ 14	
Reflections collected	5965	
Independent reflections	3132 [R (int) = 0.0148]	
Data/parameters	3132/229	
Goodness-of-fit *S* on F^2^	1.071	
R indices [I>2sigma(I)]	R1 = 0.0337, wR2 = 0.0854	
R indices (all data)	R1 = 0.0364, wR2 = 0.0871	
Largest diff. peak and hole	0.750 and −0.314 e.Å^−3^	

**Table 2:** Bond lengths [Å] and angles [°] for **1**.

Co(1)–O(1)	2.0362 (14)	O(1)–Co(1)–N(2)#1	90.95 (6)
Co(1)–N(9)	2.1089 (16)	O(1)–Co(1)–N(2)	89.05 (6)
Co(1)–N(2)	2.1536 (15)	N(9)–Co(1)–N(2)	89.51 (6)
O(1)–Co(1)–O(1)#1	180.0	N(2)#1–Co(1)–N(2)	179.999 (1)
O(1)–Co(1)–N(9)	89.10 (6)	N(9)#1–Co(1)–N(2)	90.49 (6)
O(1)–Co(1)–N(9)#1	90.90 (6)	N(9)–Co(1)–N(9)#1	180.0

Symmetry transformations used to generate equivalent atoms: #1 − *x* + 1, −*y* + 1, −*z* + 1.

**Table 3:** Hydrogen bonding and stacking interactions in **1**.

D-H ⋯ A	d(D-H)	d(H ⋯ A)	d(D ⋯ A)	<(DHA)
N(1)–H(1) ⋯ Cl(1)#1	0.81	2.270	3.083	176
N(5)–H(5) ⋯ Cl(1)#2	0.84	2.240	3.0708	169
O(1)–H(11) ⋯ N(7)#3	0.80	1.990	2.7840	173
O(1)–H(12) ⋯ Cl(1)	0.77	2.380	3.4137	176

Centroids	Centroids dist (Å)	L sq planes dist (Å)		Offset (Å)

a ⋯ b#4	3.7908 (10)	3.221		1.999
c ⋯ b#5	3.5059 (11)	3.310		1.156
b ⋯ b#4	4.0389 (10)	3.385		2.203

Symmetry codes: #1 − *x*, 1 − *y*, 1 − *z*, #2 1 − *x*, −*y*, 1 − *z*, #3 *x*, 1 + *y*, −1 + *z*, #4 2 − *x*, 1 − *y*, −*z*, #5 2 − *x*, 2 − *y*, *z*

a N(1)-N(2)-N(3)-N(4)-C(7), b C(1)-C(2)-C(3)-C(4)-C(5)-C(6), c N(5)-N(6)-N(7)-N(8)-C(8)
